# A Case of Severe Disseminated Autoeczematization Secondary to Cellulitis

**DOI:** 10.7759/cureus.25310

**Published:** 2022-05-24

**Authors:** Yash V Bhagat, Merve Otles, Brittany Salmon, Roshaye Graham, Miriam Micheal

**Affiliations:** 1 Internal Medicine, University of Maryland Medical Center Midtown Campus, Baltimore, USA; 2 Internal Medicine, American University of Antigua, St. John's, ATG; 3 Internal Medicine, Howard University College of Medicine, Washington DC, USA; 4 Internal Medicine, University of Maryland School of Medicine, Baltimore, USA

**Keywords:** skin rash, itch, rash, dermatology, pustule, vesicle, methotrexate, cellulitis, autoimmune, eczema

## Abstract

Autoeczematization, the dissemination of a local eczematous reaction to a distal site, is closely associated with lower extremity edema. Our patient is a 50-year-old man with a past medical history of drug-induced lupus to hydralazine and recent bilateral cellulitis in his lower extremities. He was presented with complaints of vesicles on his palms and soles and a scaling rash that had spread over his torso, arms, and trunk. Laboratory studies found no evidence of an active rheumatological condition with complement C3 and C4 levels being normal and no anti-dsDNA, anti-histone, anti-Smith, anti-ribonucleoprotein (anti-RNP), anti-centromere, anti-neutrophil cytoplasmic antibodies (ANCA), anti-Ro, or anti-La antibodies present. Moreover, syphilis, HIV, gonorrhea, chlamydia, rickettsia antibody, and *Borrelia burgdorferi* antibody testing was negative suggesting a non-infectious etiology of the rash. Hypothesizing a dermatologic origin of the rash, a skin biopsy was performed that revealed intermittent foci of moderate hyperparakeratosis and mild hypergranulosis indicative of eczematous dermatitis. Unfortunately, treatment of the disseminated rash with 10 mg of daily oral prednisone and topical triamcinolone acetonide 0.1% ointment proved inefficient, and methotrexate therapy was advised. We posit that cellulitis, a soft tissue infection under the skin, is a potential cause of disruption of the skin barrier that leads to activation of autosensitized T cells. These activated T cells circulate to distal areas of the skin and may lead to autoeczematization. The treatment of these id reactions with corticosteroids - both topical and oral - may be insufficient at reducing dermatitis and require the application of systemic methotrexate or cyclosporine. Through this case, we demonstrate the importance of treating id reactions by stepping up the intensity of treatment due to the severity of autosensitization-driven eczema.

## Introduction

Stasis dermatitis, also known as eczematous dermatitis, is known to present with vesicles, scales, and pruritus on the lower limbs and ankles in association with chronic venous insufficiency [1]. Patients with venous stasis often develop autoeczematization or id reaction causing breakout of vesicles in close proximity to the initial site of dermatitis before widespread dissemination at more distal regions, such as the arms, feet, and trunk [2]. Biopsies of these vesicles demonstrate a form of eczema with interstitial edema and the presence of lymphocytic infiltrates such as T cells [3]. In most instances, autoeczematization that presents with vesicular eruptions primarily on the palms is associated with dermatophyte infections such as tinea pedis and capitis [4].

The underlying cause of the id reaction dictates the method of treatment. The treatment is aimed at the resolution of dermatitis in the primary area with subsequent resolution of dermatitis in the distal location. Therefore, id reactions secondary to tinea pedis resolve following antifungals, while reactions secondary to stasis dermatitis are treated with steroids [5]. This report highlights a patient with a severe id reaction secondary to chronic stasis dermatitis resistant to topical and oral steroid therapy and requiring methotrexate, systemic treatment for severe eczema.

## Case presentation

A 50-year-old male with a past medical history of drug-induced lupus to hydralazine and bilateral lower extremity cellulitis that occurred two months ago presents with complaints of vesicles on his palms and soles and a scaling rash that had spread over his body. The patient’s prior cellulitis was treated in-patient with IV vancomycin and piperacillin-tazobactam and outpatient oral doxycycline with follow-up appointments at the wound care clinic. 

**Figure 1:**
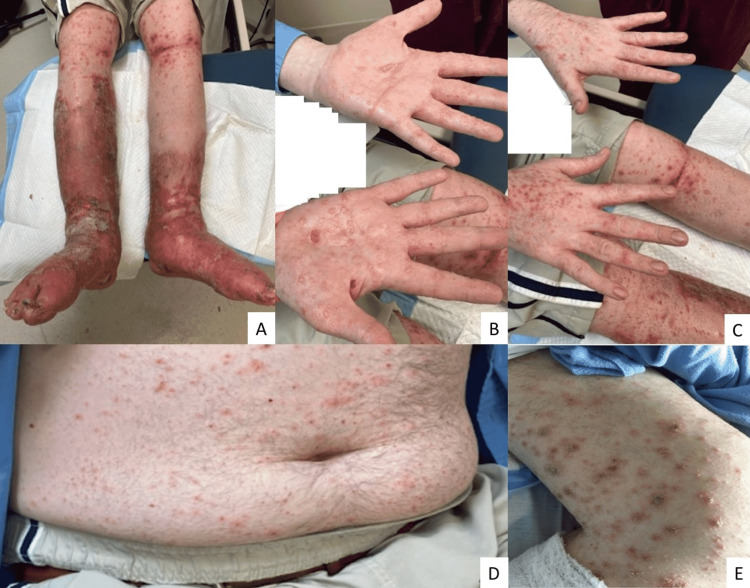
Pictures of patient's cellulitis (A), vesicular rash on palms (B), and itchy scaly rash on the dorsum of his hands (C), trunk (D), and thigh (E).

He stated that he first saw the rash begin about a week and a half ago around his left thigh above the cellulitis. The rash was distinct from his cellulitis and did not seem to cross the border of the area affected by the cellulitis (Figure 1A). About three days after, he noticed the rash had spread to the right knee and right medial thigh. A day after, the rash had spread upward to his trunk, to the dorsum of his hands, and to his back. Only thereafter, the rash was present as vesicles on his palms (Figures 1B-1E). 

The patient denies recent sick contacts, insect bites, sexual activity within the last year, and changes in medications or soap products that he uses. He is also not immunocompromised, has no history of diabetes or STDs, and is up to date with all of his childhood vaccinations.

The pustules were found only on the palms, were tense, about 2-5 mm wide, and tended to ooze purulent liquid. In comparison, the rash was scaly, had formed scabs, was itchy, and had a central head with distinct singular round units that did not overlap. The patient’s mucosa, face, scalp, groin, pelvis, perineum, and buttocks were spared from the rash and vesicles. The rash had a negative Nikolsky sign and was present in various stages of healing. 

The patient's lab work, including complete blood count (CBC), comprehensive metabolic panel (CMP), and vitals, was unremarkable. Blood cultures were drawn and empiric antibiotics (vancomycin, piperacillin-tazobactam, and valacyclovir) were started. Vancomycin and piperacillin-tazobactam were discontinued after three days and valacyclovir after 10 days due to no leukocytosis, fever, or resolution of symptoms. The patient’s serum lactate, c-reactive protein (CRP), and erythrocyte sedimentation rate (ESR) were elevated (Table 1). Rheumatologic serology revealed positive anti-nuclear antibody (ANA) titers, but normal C3 and C4 complement levels. Moreover, several antibody assays were negative (Table 1). Infectious disease testing with rapid plasma reagin (RPR), HIV antibody, gonorrhea and chlamydia polymerase chain reaction (PCR), Rickettsia antibody, and *Borrelia burgdorferi* antibody were also negative. After ruling out rheumatic and infectious etiologies, a skin punch biopsy was ordered out-patient.

**Table 1:** Laboratory tests performed on blood drawn from the patient to assess for rheumatic and infectious etiologies of the rash. anti-RNP: anti-ribonucleoprotein; ANCA: anti-neutrophil cytoplasmic antibodies; ANA: antinuclear antibody; RPR: rapid plasma regain; PCR: polymerase chain reaction

Tests performed	Result	Reference range
Lactate	High; 2.8 mmol/L	0.5-2.2 mmol/L
C-reactive protein	High; 1.3 mg/dL	<1.0 mg/dL
Erythrocyte sedimentation rate	High; 37 mm/h	1-13 mm/h for males
ANA antibodies	Positive	<1:160
C3 complement	Normal	80-160 mg/dL
C4 complement	Normal	16-48 mg/dL
Anti-dsDNA antibodies	Negative	<10 AU/mL
Anti-histone antibodies	Negative	<1 AU/mL
Anti-Smith antibodies	Negative	<7 AU/mL
Anti-RNP antibodies	Negative	<20 AU/mL
Anti-centromere antibodies	Negative	<30 AU/mL
ANCA antibodies	Negative	<20 AU/mL
Anti-Ro antibodies	Negative	<1 AU/mL
Anti-La antibodies	Negative	<1 AU/mL
RPR	Nonreactive	Nonreactive
HIV antibody	Nonreactive	Nonreactive
Gonorrhea and chlamydia PCR	Nonreactive	Nonreactive
Rickettsia IgG antibody	Nonreactive	Nonreactive
*Borrelia burgdorferi* antibody	Nonreactive	Nonreactive

Skin biopsy showed that the epidermis had mild acanthosis, mild spongiosis, mild hypergranulosis, and intermittent foci of mild to moderate hyperparakeratosis. There were focal minimal mounded patterns of hyperparakeratosis and focal intracorneal serum entrapment. The dermis showed a mild superficial perivascular and mildly interstitial infiltrate of lymphocytes, eosinophils, and plasma cells. Deeper levels showed more prominent intracorneal serum entrapment with focal sparse intracorneal neutrophils and more conspicuous plasma cells. Periodic acid-Schiff (PAS) staining is negative for fungal organisms or hyphae and spirochetes were not identified on immunohistochemical staining. The microscopic findings were suggestive of eczematous dermatitis. The patient was prescribed prednisone 10 mg once a day, clobetasol ointment for his hands, triamcinolone 0.1% ointment for the rest of the body, and doxepin 25 mg nightly. After a month, the patient reported only minimal improvement in his hands, abdomen, and upper extremities with PO prednisone and topical steroids; however, the itchy rash was still present on his bilateral lower extremities. The patient was counseled on the administration of methotrexate as a systemic line of treatment.

## Discussion

We presented a case of autoeczematization secondary to stasis dermatitis triggered by a recent episode of bilateral lower extremity cellulitis. Our patient presented with a rash following bilateral lower extremity cellulitis which first began on the lower extremities and then spread non-contiguously to cover most of his body. 

Patients with chronic stasis dermatitis are at a 37% lifetime risk of developing autoeczematization later in the course of the disease when compared to the general population [4,6]. We postulate that stasis dermatitis causes a reaction involving T cells in the absence of interferon signaling, an autoeczematization reaction. Immunologically, this T cell reaction is secondary to skin inflammation and cellular damage caused by stasis dermatitis which leads to the formation of keratinocyte antigens that are autoreactive. A study by Fehr et al. postulated that proteins derived from keratinocytes behaved like antigens which caused an autoreaction involving T cells [7,8]. Other studies demonstrated evidence of a similar reaction in a patient with hypostatic dermatitis, wherein, an elevated level of activated helper T cells was noted despite no production of interferon from peripheral mononuclear leukocytes [9]. Bertoli et al. state that these antigenic keratinocytes are able to produce cytokines - thymic stromal erythropoietin, IL-25, and IL-33 - that lead to activation of T-cells that travel to distant sites and cause an id reaction [4]. 

After investigation to rule out infectious and rheumatologic etiologies with similar presentation, our biopsy demonstrated that the severe rash in our patient was an autoeczematization reaction. Equivocal findings on biopsy have been reported by Bertoli et al. showing psoriasiform spongiotic dermatitis with mononuclear cells, eosinophils and lymphohistiocytic infiltrates [4]. The histopathological findings from our skin biopsy align with our theory that the distant rash is T cell mediated.

Identification of T cell-mediated processes as the pathology underlying autoeczematization has helped tailor treatment. A study on dupilumab, a monoclonal antibody inhibitor of IL-4 receptor, as a treatment for autoeczematization found that this treatment shifted the T cell-mediated reaction towards Th1 dominance, rather than Th2, causing a subsequent resolution of the autoeczematous rash and development of a psoriatiform rash that was different from the initial autoeczematous rash [6]. This finding guides the choice of methotrexate as a treatment. Methotrexate is hypothesized to induce apoptosis of activated T lymphocytes via a Th2-mediated mechanism. Studies by Genestier et al. showed dose-dependent inhibition of cell proliferation and apoptosis of activated peripheral T cells by methotrexate, sparing nonactivated T cells in vitro [10]. A study comparing methotrexate treatment in patients with recalcitrant eczema versus folic acid treatment found a statistically significant reduction in eczema post methotrexate treatment. The study covered many eczematous presentations, such as atopic dermatitis, allergic contact dermatitis, idiopathic pompholyx, seborrheic eczema, and lichen simplex chronicus, which they posit to be T cell-mediated and concluded that methotrexate is a drug of choice in treatment resistant eczema, regardless of its etiopathogenesis [11]. 

We do also encourage early treatment of the inciting factor, in this case, the primary venous stasis, which could lead to improvement of the distal id reaction, a finding seen with the use of compression stockings [4]. Treatment of the stasis dermatitis with prednisone, and at distant sites with clobetasol ointment, triamcinolone ointment, and doxepin also provided initial improvement but could prove futile in cases of treatment-resistant eczema, as with our patient. 

Further studies should be done to localize markers of T cells from biopsies of the distal rash [4]. Other studies have identified elevated cell surface expressions of HLD-DR antigen and IL-2R on the surface of T cells in autoeczematization reactions that were subsequently reduced after treatment and resolution of symptoms [12]. Of note, this response was only seen in autoeczematization secondary to stasis dermatitis and not in autoeczematization secondary to psoriasis which aligns with our hypothesis [12]. We suggest that HLA-DR antigen and IL-2R which were identified using fluorescent antibodies in the above study could be utilized and therefore could prove to be a standard mechanism by which we diagnose these rare episodes of autoeczematization in the future cases.

## Conclusions

In conclusion, we present a severe case of autoeczematization that was difficult to diagnose, needed lengthy workup to rule out other causes, and harder to treat, requiring stepping-up of medications from topic and oral prednisone to methotrexate. We hope that sharing this case will make clinicians aware of the varied presentation of disseminated eczema in the future, and inspire rapid diagnosis and treatment of such cases.
